# Differences between reference intervals of blood counts of Brazilian adults with and without sickle cell trait according to laboratory tests from the National Health Survey

**DOI:** 10.1590/1980-549720230003.supl.1

**Published:** 2023-04-21

**Authors:** Ana Carolina Micheletti Gomide Nogueira de Sá, Alanna Gomes da Silva, Crizian Saar Gomes, Antonio Tolentino Nogueira de Sá, Deborah Carvalho Malta

**Affiliations:** I Universidade Federal de Minas Gerais, Nursing School, Graduate Program in Nursing – Belo Horizonte (MG), Brazil.; II Universidade Federal de Minas Gerais, Nursing School, Department of Maternal-Child and Public Health Nursing, Graduate Program in Nursing – Belo Horizonte (MG), Brazil.; III Universidade Federal de Minas Gerais, Medical School, Graduate Program in Public Health – Belo Horizonte (MG), Brazil.; IV Universidade Federal de Minas Gerais, Medical School, Graduate Program in Sciences Applied to Adult Health, Hospital das Clínicas, and Department of Occupational Health Assistance – Belo Horizonte (MG), Brazil.; V Faculdade de Ciências Médicas de Minas Gerais, Medical Clinic Department – Belo Horizonte (MG), Brazil.; VI Universidade Federal de Minas Gerais, Nursing School, Department of Maternal-Child and Public Health Nursing – Belo Horizonte (MG), Brasil.

**Keywords:** Health surveys, Reference values, Blood cell count, Sickle cell trait, Brazil

## Abstract

**Objective:**

To compare reference intervals (RI) of blood counts of Brazilian adults with and without sickle cell trait (SCT).

**Methods:**

Cross-sectional study, based on the National Health Survey, 2014-2015, composed of 8,952 individuals. The sample of patients with SCT was composed of 234 adults. The RIs of adults with and without SCT were compared in the study “Reference values for laboratory tests of blood count in the Brazilian adult population: National Health Survey”, by Rosenfeld et al. (2019). The parametric method and the Student's t test were used for comparison (p≤0.05).

**Results:**

There were statistically significant differences between RIs of adults with and without SCT as far as sex is concerned for hemoglobin, MCV, MCH, MCHC, white blood cells, absolute lymphocytes, mean platelet volume and RDW; At all ages, for white blood cells and RDW in men and for MCV, MCH, MCHC, mean platelet volume and RDW in women; Between 18 to 59 years, for MCH, MCV, MCHC, neutrophils, lymphocytes and platelets in men and in women for lymphocytes, red blood cells, white blood cells, neutrophils, eosinophils, monocytes and platelets; From 60 years old on, for hemoglobin and hematocrit in men and in women for hematocrit, white blood cells, neutrophils and platelets; In white, black and brown people for white blood cells, neutrophils and platelets (p<0.05).

**Conclusion:**

Brazilian adults with SCT had lower counts of hemoglobin, MCV, MCH, MCHC, white blood cells and higher RDW than without SCT. The results show the importance of genetic counseling and further research to support the proper management of this condition in Brazil.

## INTRODUCTION

Sickle cell trait (SCT) is a condition described as usually asymptomatic^1,2^. Its etiology stems from the presence of sickle cell hemoglobin (HbS), the result of a mutation after the replacement of A by T in codon 6 of the hemoglobin beta chain. As a consequence, glutamic acid changes into the amino acid valine. Valine-type hemoglobin leads to the sickling of red blood cells when exposed to low oxygen threshold^1^. Affected individuals inherit the hemoglobin A (HbA) and HbS genes from their parents. Thus, they are heterozygous with an abnormal allele of the hemoglobin beta gene^1^.

The importance of screening^3^ and genetic counseling in people with SCT is highlighted as a means to clarify people about the risk of having children with sickle cell disease (SCD), since it is inherited through an autosomal recessive gene from both parents^2^. In SCD, individuals have two copies of the beta globin variant and the primary hemoglobin present in their red blood cells (HbS), making them homozygous (HbSS)^4^. The morbidity and mortality and severity of SCD is associated with microvasculature occlusions with tissue ischemia, leading to pain crises; organic lesions with functional asplenia; cerebral vasculopathy; kidney, lung and heart failure^4,5^.

Although SCT is commonly asymptomatic, there is evidence of cases of sudden death^2^ in affected people. Such cases happen because of complications from hypoxia, including sudden death^6^, hematuria, hyposthenuria, pulmonary embolism, splenic infarction^2,6^, urinary tract infection in women, end-stage kidney disease, polycystic kidney disease, heart attack, stroke, eye problems, and lower limb ulcers^4^.

SCT is widely distributed continental-wise^7^, being more frequent in people of African descent^1,2,8^. It is estimated that there are 300 million people with SCT around the world^1,3^, with the highest prevalence in Equatorial Africa, Arabia, India, Israel, Turkey, Greece and Italy, reaching a 50% prevalence in certain regions of these countries^7^. In Brazil, the prevalence varies between 2 and 8%^7^. Studies with data from the National Health Survey (PNS – *Pesquisa Nacional de Saúde*), between 2014 and 2015, identified a prevalence of 2.49% in adults^9^.

SCD, on the other hand, is among the genetic pathologies of greatest epidemiological importance in Brazil and in other nations^2^, considered as a public health problem by the World Health Organization (WHO)^3^. Worldwide, in 2010, SCD affected 305,800 newborns, and an increase is expected to 404,200 by 2050^10^. In Brazil, the prevalence ranges from 1.1 to 9.8% and from 0.8 to 60 per 100,000 live births in different regions of the country^2^.

Changes in hematological parameters have been identified in people with SCD^8,11^. In people with SCT, although hematological parameters are usually described as normal or without important laboratory alterations^4^, this condition can lead to vaso-occlusion due to rigid erythrocytes in the face of pathological processes that cause hypoxia, acidosis, dehydration, hyperosmolality or hypothermia^11^.

Differences in the hematological parameters of people with SCT were found in populations from other countries, which means that the reference intervals (RI) of blood counts may suffer geographic and ethnic influences^3,8^. However, studies on the hematological profile of patients with SCT in Brazil are scarce. This study steps forward by providing, in an unprecedented way, through PNS exams, the hematological parameters of adults with SCT and the differences found in parameters of adults without SCT in the country. Our findings may contribute to support the handling of SCT in Brazil.

Thus, this study aimed to compare the RI of blood counts of Brazilian adults with and without SCT.

## METHODS

### Study Design

Cross-sectional study with data from laboratory tests of the PNS carried between 2014 and 2015.

### Context and data source

The PNS is a national household-based survey carried out by the Brazilian Institute of Geography and Statistics (IBGE – *Instituto Brasileiro de Geografia e Estatística*), in partnership with the Ministry of Health^12^.

The PNS 2013 used probabilistic sampling by clusters in three stages, with stratification of the primary sampling units (UPA), being selected in the first, second and third stages, respectively: census tracts; 10 to 14 households in each UPA; and one resident over the age of 18. At all stages, the selection processes were carried out by simple random sampling (SRS). Records of 64,348 households were obtained, of which 60,202 were interviews with adults. Furthermore, the collection of exams was planned in a subsample of 25% of the census sectors, being collected, between 2014 and 2015, from 8,952 adults^12^.

Due to the complex sampling design of the PNS and the unequal selection probabilities, sample weights were used^12^. In order to used the PNS laboratory database, weights were calculated by post-stratification procedures by sex, age range, race/skin color and level of education according to major region, from the total sample of the research^12^.

Laboratory samples were collected at any time of the day and without fasting. For blood count collection and hemoglobin electrophoresis, tubes with ethylenediaminetetraacetic acid (EDTA) were used. The samples were analyzed using an automatic cell analyzer^12^. Hemoglobinopathies were searched by the HPLC method, and the results of individual tests were interpreted, providing normal, homozygous or heterozygous parameters for Hemoglobin S (HbS), Hemoglobin C (HbC), Hemoglobin D (HbD) and other hemoglobinopathies^9^. More methodological details are available in other publications^12,13^.

The PNS database and questionnaires used in this study are available at: www.pns.fiocruz.br.

### Participants

Participants were adults aged 18 and over. The PNS database, composed of 8,952 individuals, was used. A total of 330 adults were excluded due to absence of test results, insufficient material, sample loss and hemolysis^9^. In this study, data from 234 adults with SCT were analyzed. Data analyzed from adults without SCT were extracted from the study by Rosenfeld et al.^14^.

### Reference Intervals

#### Intervals of adults with sickle cell trait:

The RI of blood count parameters (described below) of adults with SCT were estimated considering the lower limit (LL) as the mean −1.96 SD (standard deviation) and the upper limit (UL), the mean +1.96 SD (standard deviation).

#### Intervals of adults without sickle cell trait

For adults without SCT, the means and RI (LL and UL) of blood count parameters from the study by Rosenfeld et al.^14^ were used.

### Variables

The variables were: sociodemographic: sex (male; female); age (age range in years: 18 to 59; 60 or older); race/skin color (white; brown; black). Presence of SCT: (yes; no). Blood count parameters: red series: red blood cells (millions/mm^3^); hemoglobin (g/dL); hematocrit (%); mean corpuscular volume (MCV) (fL); mean corpuscular hemoglobin (MCH) (pg); mean corpuscular hemoglobin concentration (MCHC) (g/dL); erythrocyte distribution range (RDW) (%). White series: white blood cells (mm^3^); absolute neutrophils (mm^3^); absolute eosinophils (mm^3^); absolute basophils (mm^3^); absolute lymphocytes (mm^3^); absolute monocytes (mm^3^); platelets (μl); mean platelet volume (fL).

### Statistical analyses

The RIs were estimated by the parametric method. The sample was partitioned according to sex, age and race/skin color. For each partition, the means, SD and RI linked to the LL and UL were calculated (mean ± 1.96 SD) according to sex, age group and race/skin color. As the PNS sample was large enough and close to normal distribution, the Student's t test was used to compare RIs of adults with and without SCT in the study by Rosenfeld et al.^14^. The significance level adopted was 5%.

Data analyses were performed using the Data Analysis and Statistical Software (Stata), version 14, with the set of commands in the survey module, which considers post-stratification weights.

### Ethical Aspects

The PNS was approved by the National Research Ethics Committee of the National Health Council (Opinion No. 328,159). Adult's participation was voluntary, and confidentiality of information was guaranteed^13^.

## RESULTS

For the red series, there were statistically significant differences for some blood count parameters when comparing RIs of adults with and without SCT^14^ (p≤0.05) (Table 1). Men and women with SCT had lower mean values of hemoglobin, VCM, MCH, MCHC (Table 1) when compared to those without SCT^14^, as one can see from the means and RIs of the study by Rosenfeld et al.^14^ displayed in Table S1. For RDW, the means of men and women with SCT were higher than those without SCT^14^. Men with SCT still had lower hematocrit means than those without SCT ^14^ (Tables 1 and S1).

**Table 1:** Blood count reference intervals for adults ≥18 years old with sickle cell trait for red blood cells series by sex, National Health Survey, Brazil, 2014–2015.

Parameters	Biological sex	n*	Mean	SD	LL	UL	p†
Red blood cells (millions/mm^3^)	Male	84	5.1	0.4	4.3	5.9	0.6441
	Female	128	4.6	0.4	3.8	5.4	0.05
Hemoglobin (g/dL)	Male	84	14.5	1.3	12.0	17.0	0.0013
	Female	128	13.0	1.5	10.1	15.9	0.0004
Hematocrit (%)	Male	84	45.5	4.1	37.5	53.5	0.0254
	Female	128	40.5	3.6	33.4	47.6	0.0652
Mean corpuscular volume (fL)	Male	84	89.6	4.9	80	99.2	0.0027
	Female	128	88.0	5.1	78	98	<0.01
Mean corpuscular hemoglobin (pg)	Male	84	28.9	1.7	25.6	32.2	<0.01
	Female	128	28.3	1.7	25.0	31.5	<0.01
Mean corpuscular hemoglobin concentration (g/dL)	Male	84	32.2	1.1	30.0	34.4	0.0029
	Female	128	32.1	1.0	30.1	34.0	0.0007
Erythrocyte Distribution Width (RDW) (%)	Male	84	13.9	1.0	11.9	15.8	0.0001
	Female	128	14.3	1.4	11.7	17.0	<0.01

SD: standard deviation; LL: lower limit; UL: upper limit.

* The sample of adults with sickle cell trait was composed by 234 participants, but missing data were not presented;

† Comparison of blood count parameters in adults with and without sickle cell trait from the study by Rosenfeld et al.^14^ using Student's t test (statistically significant differences = p≤0.05).

Regarding the white series, for both sexes, statistically significant differences were observed in the RIs of some parameters of the blood count of men and women with SCT compared to those without SCT^14^ (p≤0.05) (Table 2). The means of white blood cells, neutrophils, lymphocytes and platelet volume were lower in men and women with SCT than in those without SCT^14^. Women with SCT also had higher mean eosinophils and platelets values than those without SCT^14^, and men with SCT had lower mean platelets than those without SCT^14^ (Tables 2 and S1).

**Table 2:** Blood count reference intervals for adults ≥ 18 years old with sickle cell trait for the white blood cell series according to sex, National Health Survey, Brazil, 2014-2015.

Parameters	Biological sex	n*	Mean	SD	LL	UL	p†
White blood cells (mm^3^)	Male	75	5.374.8	1.682.2	2.077.7	8.671.9	<0.01
	Female	117	6.390.2	2.056.7	2.359.1	10.421.3	<0.01
Absolute neutrophils (mm^3^)	Male	74	2.940.4	1.373.1	249.1	5.631.7	<0.01
	Female	117	3.674.9	1.723.7	296.4	7.053.4	<0.01
Absolute eosinophils (mm^3^)	Male	74	304.4	274.4	233.4	842.2	0.3509
	Female	117	225.0	214.6	195.6	645.6	0.0189
Absolute basophils (mm^3^)	Male	74	25.5	23.4	20.3	71.4	0.1260
	Female	117	33.5	31.4	28.0	95.0	0.9750
Absolute lymphocytes (mm^3^)	Male	74	1.702.3	669.3	309.5	3.014.1	<0.01
	Female	117	2.074.4	756.9	590.9	3.557.9	<0.01
Absolute monocytes (mm^3^)	Male	74	428.0	232.6	55.8	800.1	0.8392
	Female	117	382.5	191.6	7.0	758.0	0.0518
Platelets (μl)	Male	64	197.645.2	42.288.1	114.760.5	280.529.9	<0.01
	Female	119	255.814.0	64.357.9	129.672.5	381.955.5	<0.01
Mean platelet volume (fL)	Male	64	8.8	4.1	0.8	16.8	0.042
	Female	119	8.6	3.8	1.2	16.0	<0.01

SD: standard deviation; LL: lower limit; UL: upper limit.

* The sample of adults with sickle cell trait was composed by 234 participants, but missing data were not presented;

† Comparison of blood count parameters in adults with and without sickle cell trait from the study by Rosenfeld et al.^14^ using Student's t test (statistically significant differences = p≤0.05).

With regard to age, for the red series, there were statistically significant differences in the RIs of men with SCT compared to those without SCT^14^ (p≤0.05) (Table S2). Means were lower between 18- and 59-year-olds for MCH, MCV and MCHC, and from 60 years onwards for hemoglobin, hematocrit and MCH in men with SCT than in those without SCT^14^. RDW means were higher in men aged between 18 and 59 years old with SCT than in those without SCT^14^. The means of hemoglobin, MCV, MCH and MCHC in women aged between 18 and 59 years and 60 years or older were lower in those with SCT than in those without SCT^14^. Women with SCT aged 60 years or older had lower hematocrit means compared to those without SCT^14^. Higher mean red blood cells and RDW were found in women aged 18 to 59 years with SCT compared to those without SCT^14^ (Tables S2 and S3).

For the white series, there were statistically significant differences in the RIs of adults with and without SCT^14^ when stratified by age (p≤0.05) (Table S4). Mean white blood cell values were lower in men with SCT than in those without SCT^14^ aged between 18 and 59 years and 60 and older. Lower means of neutrophils, lymphocytes, platelets and platelet volume were identified in men with SCT when compared to those without SCT^14^ in the two age groups. For women with SCT, the mean platelet volumes were lower in both age groups than for those without SCT^14^ and aged 60 and older, women with SCT had lower mean white blood cells, neutrophils and platelets compared to those without SCT^14^. Also in women with SCT compared to those without SCT^14^, higher means of white blood cells, neutrophils, eosinophils, monocytes and platelets were found in the age group 18-59 years (p≤0.05) (Table S3 and S4).

There were differences when comparing the RIs of some blood count parameters between men and women with and without SCT^14^ of white, brown and black skin for hemoglobin, white blood cells, neutrophils and platelets (p≤0.05) (Table S5). The means were slightly lower for both sexes for hemoglobin in brown adults with SCT than in those without SCT^14^. In white, black and brown men with SCT, the mean white blood cells, neutrophils and platelets were lower than in those without SCT^14^. White women with SCT had lower means of white blood cells and neutrophils than those without SCT^14^, and black women had lower platelet means. In black and brown women with SCT, higher mean values of white blood cells and neutrophils were found, while for white and brown women, mean values of platelets were higher compared to those without SCT^14^ (Tables S5 and S6).

## DISCUSSION

This study assessed for the first time, using laboratory PNS data, the differences in hematological RI of Brazilian adults with and without SCT^14^. The findings show lower counts for hemoglobin, MCV, MCH, MCHC, white blood cells, lymphocytes and mean platelet volume, and higher counts for RDW in adults with SCT compared to those without SCT^14^, for both sexes. When stratifying according to sex and age, differences were identified in some parameters of white and red series when comparing adults with and without SCT^14^. Furthermore, differences were found in the RI of white, black and brown adults with SCT for white blood cells, neutrophils and platelets, and also in the brown adults for hemoglobin, when compared to those without SCT^14^. Such findings show the relevance of knowing the hematological profile of people with SCT for an adequate management of this condition in the country.

Bearing in mind that hematological parameters of people with SCT usually do not show important laboratory alterations^4^, the differences found in the RI of some parameters of the blood count of Brazilian adults with and without SCT^14^ point to the need for further studies to better understand the clinical implications in people with this condition.

In this study, the results found for the blood count RIs in adults with and without SCT^14^, according to sex, were consistent with a study carried out in Ghana, in which individuals with and without SCD^8^ were compared. With regard to the red blood count series, in Brazilian adults of both sexes with SCT, the altered values of hemoglobin, MCHC and MCV indicate anemia^15–18^, microcytosis^17–19^ and hypochromia^16,17^, unlike adults without SCT, whose reference values were unchanged^14^. Furthermore, low MCHC values in adults with SCT may be associated with iron deficiency anemia^20^ or thalassemias^21^. Other findings in these people with SCT refer to hypochromic microcytic anemia with high RDW, which is also characteristic of lack of iron from nutritional deficit, deficiency in absorption due to gastrointestinal alterations and chronic loss of this micronutrient^22^, as evidenced by the UL of RDW.

This finding is alarming due to the possibility of malnutrition in people with SCT in Brazil, especially when taking into account that chronic hemolysis results in greater availability of iron, and its deficiency is unlikely in SCD^23,24^. This highlights the need to intensify actions in the field of food security in Brazil, with special attention to the most vulnerable groups^16^. This situation was aggravated by the COVID-19 pandemic, which put Brazil back on the hunger map^25^, and may have contributed to the worsening of this scenario in people with SCT and SCD. In compliance with the national and international pacts made by Brazil to reduce iron deficiency anemia by 2030, this should be one of the priorities in public health^16^.

Blood count analyses by age followed similar patterns of analyses by sex, occurring in men and women with SCT of all ages, when compared to those without SCT^14^, alterations in the RI of MCV, MCH and MCHC. Possible explanations are the conditions of access health and food, as well as health assistance, once the clinical variability of SCD is influenced by social and economic factors^26^—which may also be happening for SCT. Other possible explanations for the lower values of MCV, MCH and MCHC observed in adults with SCT are the effects of anemia of chronic disease, higher risk of infections and the degree of hemolysis in which such parameters are reduced^24^.

When analyzing the white blood count series according to sex, with regard to white blood cells and neutrophils, what draws attention are the even lower mean values found in Brazilian adults with SCT compared to those without SCT. In the analyses by age, the presence of leukopenia^27^ is noted by the LL in all age groups and in both sexes, and the presence of leukocytosis^27^ in women aged between 18 and 59 years identified by the UL. The literature describes that the decline in leukocytes is related to aging as a result of immune response to the extent that exposure to pathogens occurs^28,29^. Leukopenia can also result from viral and bacterial infections and medication use, and it is interchangeable with neutropenia^30^, as one can see by the LL of neutrophil found in Brazilian men and even women with SCT, whose mean values were slightly higher than in those without SCT^14^. Managing these situations requires identification of cause and effective therapy^30^. This finding possibly denotes greater clinical fragility of Brazilian adults with SCT exposed to malnutrition and infections.

As for the higher white blood cell count in Brazilian women with SCT aged between 18 and 59 years, when compared to those without SCT^14^ identified by the UL, similar results were found in Nigeria, but in the SCD.^24^ It is also worth mentioning that, in people with SCD, leukocytosis can occur due to autosplenectomy resulting from occlusion of splenic vessels, increasing vulnerability to infections^24^. Leukocytosis in SCD indicates a poor prognosis, being a risk factor for early death, stroke and acute chest syndrome, while a reduction in neutrophil counts is associated with a good prognosis^24^. There is evidence to suggest that the phagocytic function of neutrophils may be reduced in individuals with SCD, implying that their phagocytic competence affects the clinical severity of the disease^24,31^. All explanations lack empirical and theoretical evidence, so they need to be further investigated in adults with SCT.

As for the significant differences found in platelets, when stratified by sex and age, the highest mean values in women with SCT aged between 18 and 59 years compared to those without SCT^14^ may stem from thrombocytosis, attributed to hemolytic anemia and autosplenectomy associated with SCD^24,32^. It is well established in the literature that thrombocytosis is associated with SCD and several types of anemia^24,32^. The lower mean levels of platelets in men with SCT compared to those without SCT^14^ and aged between 18 and 59 years may result from alterations in components of hemostasis, including platelet function, procoagulant mechanisms, anticoagulants and the fibrinolytic system in SCD^33^. The hypercoagulable state present in people with HbS is related to qualitative changes in platelets^33^, which would possibly explain these findings.

The other hematological alterations found in adults with SCT compared to those without SCT^14^, in women aged between 18 and 59 years for eosinophils and monocytes, and hematocrit after 60 years old, as well as those found in men aged 18 to 59 years old for lymphocytes and hemoglobin, and hematocrit at 60 years or older, can be attributed to behavioral, genetic, nutritional, socioeconomic factors, as well as exposure to allergens, infections and parasitic loads^34^.

In the present study, there were substantial differences in the hematological RI of Brazilian adults with and without SCT^14^ according to skin color. Anemia identified by lower hemoglobin levels in brown adults with SCT compared to those without SCT^14^ deserves to be investigated, given that conditions associated with anemia differ by ethnicity^35^. Although racial differences in hematological parameters may be due to heredity and chronic diseases, socioeconomic factors differ according to ethnicity as well^35^. The literature reports the lack of studies that explore racial differences in hematological parameters^35^. Black populations are underrepresented in epidemiological studies on the subject^35^, and we also identified the lack of investigations in the brown population. This is an important point of discussion, given that the Brazilian population is characterized by miscegenation, different ethnicities and social/economic segments^14^, which has implications for the epidemiology of SCT and SCD in the country^36^.

This study had limitations inherent to cross-sectional studies, such as the impossibility of establishing a causal relationship; some findings in blood count parameters may reflect lifestyle and treatment. However, the hematological profile of Brazilian patients with SCT has not been studied extensively in recent years, and this is the first study with this purpose. Given the representative sample of the Brazilian population, the generalization of results is relatively safe for national estimates. It was not possible to identify people with SCD due to unavailability in the PNS database; laboratory alterations may lead to underdiagnosis of SCD in the country. Thus, our results must be interpreted with caution. Furthermore, there is controversy in the literature whether SCT is a benign state or an intermediate phenotype of the disease, and at the same time, we lack studies on this condition and its complications, which could be underestimated^37^. Evidence suggests that SCT may not be a completely benign state nor a disease, but a risk factor for adverse events resulting from the interaction between genetic and environmental influences^38^.

This study showed differences between the RI of Brazilian adults with and without SCT^14^. These results point out the importance of neonatal screening to identify SCT, genetic counseling and the need for research that characterizes the consequences and risks to health, to support the control and prevention of diseases. The detection of individuals with heterozygous hemoglobinopathies such as SCT is extremely relevant in terms of public health^39^ for its adequate management in Brazil.
