# Erratum

**DOI:** 10.1590/1980-549720230003.supl.1erratum

**Published:** 2024-09-30

In the manuscript "**Differences between reference intervals of blood counts of Brazilian adults with and without sickle cell trait according to laboratory tests from the National Health Survey**", DOI: https://doi.org/10.1590/1980-549720230003.supl.1, published in the Rev Bras Epidemiol. 2023; 26(Suppl 1): e230003.supl.1:

**On page 1 it was included:**

SCIENTIFIC EDITOR: Márcia Furquim de Almeida http://orcid.org/0000-0003-0052-1888

