# Supplementary material for: Differences between reference intervals of blood counts of Brazilian adults with and without sickle cell trait according to laboratory tests from the National Health Survey
Source: Rev Bras Epidemiol. 2023 Apr 21;26(Suppl 1):e230003. doi: 10.1590/1980-549720230003.supl.1 (PMC10176731; doi:10.1590/1980-549720230003.supl.1)
Supplement: Supplementary file 1 [file 1980-5497-rbepid-26-suppl1-e230003-suppl1.docx]

**Tabela S1 – Intervalos de referência de hemograma de adultos ≥ 18 anos sem traço falciforme para as séries vermelha e branca segundo sexo, Pesquisa Nacional de Saúde, Brasil, 2014-2015**

|  | **Sexo masculino** | | | **Sexo Feminino** | | |
| --- | --- | --- | --- | --- | --- | --- |
| **Parâmetros** | **Média** | **LI** | **LS** | **Média** | **LI** | **LS** |
| ***Série Vermelha*** | | | | | | |
| Hemoglobina (g/dL) | 14,9 | 13,0 | 16,9 | 13,2 | 11,5 | 14,9 |
| Hematócrito (%) | 45,8 | 39,7 | 52 | -- | | |
| Volume Corpuscular Médio (fL) | 91,2 | 81,8 | 100,6 | 90,6 | 81 | 100,2 |
| Hemoglobina Corpuscular Média (pg) | 29,8 | 26,9 | 32,6 | 29,4 | 26,3 | 32,4 |
| Concentração de Hemoglobina Corpuscular Média (g/dL) | 32,6 | 30,6 | 34,6 | 32,4 | 30,5 | 34,3 |
| Amplitude de distribuição dos eritrócitos (RDW) (%) | 13,6 | 12 | 15,3 | 13,7 | 11,9 | 15,5 |
| ***Série Branca*** | | | | | | |
| Glóbulos Brancos (mm^3^) | 6.142 | 2.843 | 9.440 | 6.426 | 2.883 | 9.969 |
| Neutrófilos Absolutos (mm^3^) | 3.273 | 576 | 5.971 | 3.543 | 612 | 6.474 |
| Eosinófilos Absolutos (mm^3^) | -- | | | 210 | 0 | 550 |
| Linfócitos Absolutos (mm^3^) | 2.045 | 720 | 3.370 | 2.105 | 796 | 3.414 |
| Plaquetas (μl) | 213.975 | 128.177 | 299.774 | 239.325 | 135.606 | 343.044 |
| Volume plaquetário Médio (fL) | 10 | 8 | 12 | 10 | 8 | 13 |

DP: desvio padrão. LI: limite inferior. LS: limite superior. Os valores apresentados nesta tabela foram extraídos do estudo de Rosenfeld *et al*.^14^. Foram apresentados apenas parâmetros de hemograma em que houve diferenças estatisticamente significativas (valor-p ≤ 0,05) na comparação de adultos com e sem^14^. -- Não houve diferenças estatisticamente significativas (valor-p > 0,05).

**Tabela S2 – Intervalos de referência de hemograma de adultos ≥ 18 anos com traço falciforme para a série vermelha segundo idade e sexo, Pesquisa Nacional de Saúde, Brasil, 2014-2015**

| **Parâmetros** | **Idade** | **n*** | **Média** | **DP** | **LI** | **LS** | **p**** |
| --- | --- | --- | --- | --- | --- | --- | --- |
| ***Sexo masculino*** | | | | | | | |
| Glóbulos vermelhos (milhões/mm^3^) | 18-59 | 60 | 5,1 | 0,4 | 4,3 | 5,9 | 0,5408 |
|  | 60 ou mais | 24 | 4,8 | 0,3 | 4,2 | 5,4 | 0,4489 |
| Hemoglobina (g/dL) | 18-59 | 60 | 14,9 | 1,3 | 12,4 | 17,4 | 0,0558 |
|  | 60 ou mais | 24 | 14,0 | 1,3 | 11,5 | 16,5 | 0,0066 |
| Hematócrito (%) | 18-59 | 60 | 46,2 | 4,0 | 38,4 | 54,0 | 0,4170 |
|  | 60 ou mais | 24 | 43,4 | 3,6 | 36,3 | 40,5 | 0,0189 |
| Volume Corpuscular Médio (fL) | 18-59 | 60 | 89,3 | 4,8 | 79,9 | 98,7 | 0,0170 |
|  | 60 ou mais | 24 | 90,4 | 5,2 | 80,2 | 100,6 | 0,0341 |
| Hemoglobina Corpuscular Média (pg) | 18-59 | 60 | 28,8 | 1,7 | 25,5 | 32,1 | <0,01 |
|  | 60 ou mais | 24 | 29,2 | 1,7 | 25,9 | 32,5 | 0,0015 |
| Concentração de Hemoglobina Corpuscular Média (g/dL) | 18-59 | 60 | 32,3 | 1,1 | 30,1 | 34,4 | 0,0173 |
|  | 60 ou mais | 24 | 32,3 | 1,0 | 30,3 | 34,3 | <0,01 |
| Amplitude de distribuição dos eritrócitos (RDW) (%) | 18-59 | 60 | 13,8 | 1,0 | 11,2 | 15,8 | 0,0068 |
|  | 60 ou mais | 24 | 14,2 | 1,0 | 12,2 | 16,2 | 0,1223 |
| ***Sexo feminino*** | | | | | | | |
| Glóbulos vermelhos (milhões/mm^3^) | 18-59 | 100 | 4,6 | 0,4 | 3,8 | 5,4 | 0,0028 |
|  | 60 ou mais | 28 | 4,4 | 0,4 | 3,6 | 5,2 | 0,3348 |

| Hemoglobina (g/dL) | 18-59 | 100 | 13,1 | 1,2 | 10,7 | 15,5 | 0,0100 |
| --- | --- | --- | --- | --- | --- | --- | --- |
|  | 60 ou mais | 28 | 12,6 | 1,5 | 9,7 | 15,5 | 0,0139 |
| Hematócrito (%) | 18-59 | 100 | 40,7 | 3,5 | 33,8 | 47,6 | 0,3815 |
|  | 60 ou mais | 28 | 39,2 | 3,8 | 31,6 | 46,6 | 0,0246 |
| Volume Corpuscular Médio (fL) | 18-59 | 100 | 88,0 | 5,1 | 78,0 | 98,0 | <0,01 |
|  | 60 ou mais | 28 | 88,4 | 4,8 | 79,0 | 97,8 | 0,0102 |
| Hemoglobina Corpuscular  Média (pg) | 18-59 | 100 | 28,3 | 1,7 | 25,0 | 31,6 | <0,01 |
|  | 60 ou mais | 28 | 28,3 | 1,5 | 25,4 | 31,2 | <0,01 |
| Concentração de Hemoglobina Corpuscular  Média (g/dL) | 18-59 | 100 | 32,2 | 1,1 | 30,0 | 34,4 | 0,0020 |
|  | 60 ou mais | 28 | 32,1 | 0,8 | 30,5 | 33,7 | <0,01 |
| Amplitude de distribuição dos eritrócitos (RDW) (%) | 18-59 | 100 | 14,3 | 1,4 | 11,7 | 17,0 | <0,01 |
|  | 60 ou mais | 28 | 14,3 | 1,2 | 11,9 | 16,7 | 0,0899 |

DP: desvio padrão. LI: limite inferior. LS: limite superior. *O valor total da amostra de adultos com traço falciforme é 234 participantes, porém dados faltantes não foram apresentados. **Comparação dos parâmetros de hemograma de adultos com e sem traço falciforme do estudo de Rosenfeld *et al*.^14^ pelo teste t de Student (diferenças estatisticamente significativas valor-p ≤ 0,05).

**Tabela S3 – Intervalos de referência de hemograma de adultos ≥ 18 anos sem traço falciforme^14^ para as séries vermelha e branca segundo idade e sexo, Pesquisa Nacional de Saúde, Brasil, 2014-2015**

|  |  | **Sexo masculino** | | | | **Sexo Feminino** | | |
| --- | --- | --- | --- | --- | --- | --- | --- | --- |
| **Parâmetros** | **Idade** | **Média** | **LI** | **LS** | **Média** | | **LI** | **LS** |
| ***Série Vermelha*** | | | | | | | | |
| Glóbulos vermelhos (milhões/mm^3^) | 18-59 |  | -- |  | 4,5 | | 3,9 | 5,1 |
|  | 60 ou mais |  | -- |  |  | | -- |  |
| Hemoglobina (g/dL) | 18-59 |  | -- |  | 13,2 | | 11,5 | 14,8 |
|  | 60 ou mais | 14,5 | 12,3 | 16,8 | 13,2 | | 11,3 | 15,1 |
| Hematócrito (%) | 18-59 |  | -- |  |  | | -- |  |
|  | 60 ou mais | 44,7 | 38,0 | 51,4 | 40,9 | | 35,1 | 46,7 |
| Volume Corpuscular Médio (fL) | 18-59 | 90,9 | 81,5 | 100,2 | 90,5 | | 81,0 | 100,1 |
|  | 60 ou mais |  | -- |  | 91,0 | | 81,2 | 100,7 |
| Hemoglobina Corpuscular Média (pg) | 18-59 | 29,7 | 26,9 | 32,5 | 29,3 | | 26,3 | 32,3 |
|  | 60 ou mais | 30,3 | 27,6 | 33,1 | 29,5 | | 26,3 | 32,6 |
| Concentração de Hemoglobina Corpuscular Média (g/dL) | 18-59 | 32,6 | 30,6 | 34,6 | 32,4 | | 30,5 | 34,3 |
|  | 60 ou mais | 32,5 | 30,6 | 34,5 | 32,4 | | 30,5 | 34,3 |
| Amplitude de distribuição dos eritrócitos (RDW) (%) | 18-59 | 13,6 | 12,0 | 15,2 | 13,7 | | 11,9 | 15,4 |
|  | 60 ou mais |  | -- |  |  | | -- |  |
| ***Série Branca*** | | | | | | | | |
| Glóbulos Brancos (mm^3^) | 18-59 | 6.124 | 2.844 | 9.403 | 6.478 | | 2.908 | 10.047 |
|  | 60 ou mais | 6.246 | 2.818 | 9.675 | 6.197 | | 2.971 | 9.424 |

| Neutrófilos Absolutos (mm^3^) | 18-59 | 3.230 | 552 | 5.909 | 3.577 | 597 | 6.557 |
| --- | --- | --- | --- | --- | --- | --- | --- |
|  | 60 ou mais | 3.528 | 724 | 6.332 | 3.366 | 728 | 6.005 |
| Eosinófilos Absolutos (mm^3^) | 18-59 |  | -- |  | 208 | 0 | 546 |
|  | 60 ou mais |  | -- |  |  | -- |  |
| Linfócitos Absolutos (mm^3^) | 18-59 | 2.086 | 767 | 3.405 | 2.122 | 825 | 3.419 |
|  | 60 ou mais | 1.835 | 582 | 3.088 | 2.039 | 716 | 3.362 |
| Monócitos Absolutos (mm^3^) | 18-59 |  | -- |  | 353 | 19 | 688 |
|  | 60 ou mais |  | -- |  |  | -- |  |
| Plaquetas (μl) | 18-59 | 215.301 | 128.418 | 302.183 | 241.312 | 137.881 | 344.744 |
|  | 60 ou mais | 206.421 | 128.926 | 283.915 | 229.057 | 126.639 | 331.474 |
| Volume plaquetário Médio (fL) | 18-59 | 10 | 8 | 13 | 10 | 8 | 13 |
|  | 60 ou mais | 10 | 8 | 12 | 10 | 8 | 12 |

DP: desvio padrão. LI: limite inferior. LS: limite superior. Os valores apresentados nesta tabela foram extraídos do estudo de Rosenfeld *et al*.^14^. Foram apresentados apenas parâmetros de hemograma em que houve diferenças estatisticamente significativas (valor-p ≤ 0,05) na comparação de adultos com e sem^14^. -- Não houve diferenças estatisticamente significativas (valor-p > 0,05).

**Tabela S4 – Intervalos de referência de hemograma de adultos ≥ 18 anos com traço falciforme para a série branca segundo idade e sexo, Pesquisa Nacional de Saúde, Brasil, 2014-2015**

| **Parâmetros** | **Idade** | **n*** | **Média** | **DP** | **LI** | **LS** | **p**** |
| --- | --- | --- | --- | --- | --- | --- | --- |
| ***Sexo masculino*** | | | | | | | |
| Glóbulos Brancos  (mm^3^) | 18-59 | 51 | 5.300,0 | 1.715,2 | 1.938,2 | 8.661,8 | <0,01 |
|  | 60 ou mais | 24 | 5.549,8 | 1.625,6 | 2.363,6 | 8.736,0 | <0,01 |
| Neutrófilos Absolutos (mm^3^) | 18-59 | 50 | 2.719,5 | 1.409,4 | 42,9 | 5.481,9 | <0,01 |
|  | 60 ou mais | 24 | 3.450,0 | 1.159,1 | 1.178,2 | 5.721,8 | <0,01 |
| Eosinófilos Absolutos (mm^3^) | 18-59 | 50 | 320,4 | 294,2 | 256,2 | 897,0 | 0,3160 |
|  | 60 ou mais | 24 | 267,3 | 223,5 | 170,8 | 705,4 | 0,8899 |
| Basófilos Absolutos (mm^3^) | 18-59 | 50 | 25,8 | 19,9 | 13,2 | 64,8 | 0,2030 |
|  | 60 ou mais | 24 | 24,8 | 30,4 | 34,8 | 84,4 | 0,3879 |
| Linfócitos Absolutos (mm^3^) | 18-59 | 50 | 1.814,8 | 641,3 | 557,9 | 3.071,7 | <0,01 |
|  | 60 ou mais | 24 | 1.442,8 | 673,9 | 122,0 | 2.763,6 | <0,01 |
| Monócitos Absolutos (mm^3^) | 18-59 | 50 | 455,4 | 226,9 | 10,8 | 900,1 | 0,5199 |
|  | 60 ou mais | 24 | 364,8 | 238,1 | 101,9 | 564,5 | 0,1494 |
| Plaquetas (μl) | 18-59 | 46 | 201.659 | 47.032 | 109.476 | 293.842 | <0,01 |
|  | 60 ou mais | 18 | 186.588 | 22.587 | 142.317 | 230.858 | <0,01 |
| Volume plaquetário Médio (fL) | 18-59 | 46 | 8,6 | 4,4 | 0,00 | 17,2 | 0,0688 |
|  | 60 ou mais | 18 | 9,2 | 3,1 | 3,1 | 15,3 | 0,3827 |
| ***Sexo Feminino*** | | | | | | | |
| Glóbulos Brancos  (mm^3^) | 18-59 | 94 | 6.656,9 | 2.178,3 | 2.387,4 | 10.926,4 | <0,01 |
|  | 60 ou mais | 23 | 5.470,7 | 1.200,0 | 3.118,7 | 7.822,7 | <0,01 |

| Neutrófilos Absolutos mm^3^) | 18-59 | 94 | 3.819,0 | 1.837,7 | 217,1 | 7.420,9 | <0,01 |
| --- | --- | --- | --- | --- | --- | --- | --- |
|  | 60 ou mais | 23 | 3.178,2 | 1.149,3 | 925,6 | 5.430,8 | <0,01 |
| Eosinófilos Absolutos (mm^3^) | 18-59 | 94 | 239,1 | 223,0 | 198,0 | 676,2 | <0,01 |
|  | 60 ou mais | 23 | 176,5 | 178,1 | 172,6 | 525,6 | 0,4388 |
| Basófilos Absolutos (mm^3^) | 18-59 | 94 | 34,1 | 31,5 | 27,6 | 95,8 | 0,9865 |
|  | 60 ou mais | 23 | 31,2 | 31,3 | 30,1 | 92,5 | 0,7255 |
| Linfócitos Absolutos mm^3^) | 18-59 | 94 | 2.163,2 | 807,1 | 581,3 | 3.745,1 | <0,01 |
|  | 60 ou mais | 23 | 1.768,3 | 437,7 | 910,4 | 2.626,2 | <0,01 |
| Monócitos Absolutos (mm^3^) | 18-59 | 94 | 401,5 | 198,2 | 13,0 | 790,0 | 0,0155 |
|  | 60 ou mais | 23 | 316,6 | 152,2 | 18,3 | 614,9 | 0,4684 |
| Plaquetas (μl) | 18-59 | 96 | 264.491 | 62.384,3 | 142.218 | 386.764 | <0,01 |
|  | 60 ou mais | 23 | 225.382 | 63.148,2 | 101.612 | 348.152 | <0,01 |
| Volume plaquetário Médio (fL) | 18-59 | 96 | 8,7 | 3,8 | 1,3 | 16,1 | 0,0020 |
|  | 60 ou mais | 23 | 8,2 | 3,9 | 0,6 | 15,8 | 0,0287 |

DP: desvio padrão. LI: limite inferior. LS: limite superior. *O valor total da amostra de adultos com traço falciforme é 234 participantes, porém dados faltantes não foram apresentados. **Comparação dos parâmetros de hemograma de adultos com e sem traço falciforme do estudo de Rosenfeld *et al*.^14^ pelo teste t de Student (diferenças estatisticamente significativas valor-p ≤ 0,05).

**Tabela S5 – Intervalos de referência de hemograma de adultos ≥ 18 anos com traço falciforme para as séries vermelha e branca segundo raça/cor da pele e sexo, Pesquisa Nacional de Saúde, Brasil, 2014-2015**

| **Parâmetros** |  | **Sexo Masculino** | | | | | | | **Sexo Feminino** | | | | | | |
| --- | --- | --- | --- | --- | --- | --- | --- | --- | --- | --- | --- | --- | --- | --- | --- |
|  | **Raça** | **n*** | **Média** | **DP** | **LI** | **LS** | **p**** | **n*** | | **Média** | **DP** | **LI** | **LS** | **p**** |  |
| ***Série Vermelha*** | | | | | | | | | | | | | | | |
| Glóbulos vermelhos (milhões/mm^3^) | Branca | 16 | 5,3 | 0,4 | 4,5 | 6,1 | 0,1939 | 21 | | 4,6 | 0,4 | 3,8 | 5,4 | 0,6321 |  |
|  | Parda | 52 | 5,0 | 0,4 | 4,2 | 5,8 | 0,4538 | 78 | | 4,6 | 0,4 | 3,8 | 5,4 | 0,1625 |  |
|  | Preta | 15 | 5,1 | 0,6 | 3,9 | 6,3 | 0,3039 | 26 | | 4,5 | 0,4 | 3,7 | 5,3 | 0,2673 |  |
| Hemoglobina (g/dL) | Branca | 16 | 15,1 | 1,2 | 12,7 | 17,5 | 0,1291 | 21 | | 13,1 | 1,0 | 11,1 | 15,1 | 0,0500 |  |
|  | Parda | 52 | 14,5 | 1,4 | 11,8 | 17,2 | 0,0335 | 78 | | 13,0 | 1,3 | 10,5 | 15,5 | 0,0281 |  |
|  | Preta | 15 | 14,1 | 1,9 | 10,4 | 17,8 | 0,4523 | 26 | | 12,6 | 1,2 | 10,2 | 15,0 | 0,8060 |  |
| ***Série Branca*** |  |  |  |  |  |  |  |  | |  |  |  |  |  |  |
| Glóbulos Brancos  (mm^3^) | Branca | 15 | 5.770,4 | 1.489,7 | 2.850,6 | 8.690,2 | <0,01 | 21 | | 6.373,2 | 1.646,9 | 3.145,3 | 9.601,1 | <0,01 |  |
|  | Parda | 48 | 5.211,1 | 1.795,2 | 1.692,5 | 8.729,7 | <0,01 | 71 | | 6.436,1 | 2.208,8 | 2.106,9 | 10.765,3 | <0,01 |  |
|  | Preta | 11 | 5.145,6 | 1.452,2 | 2.299,3 | 7991,9 | <0,01 | 22 | | 6.245,1 | 2.081,8 | 2.164,8 | 10.325,4 | <0,01 |  |
| Neutrófilos Absolutos (mm^3^) | Branca | 15 | 3.195,2 | 953,0 | 1.327,3 | 5063,1 | <0,01 | 21 | | 3.584,7 | 1.216,0 | 1.201,3 | 5.968,1 | <0,01 |  |
|  | Parda | 47 | 2.839,9 | 1.555,9 | 209,7 | 5.889,5 | <0,01 | 71 | | 3.800,2 | 1.956,8 | 35,1 | 7.635,5 | <0,01 |  |
|  | Preta | 11 | 2.763,2 | 1.283,3 | 248,5 | 5.277,9 | <0,01 | 22 | | 3.428,7 | 1.500,4 | 487,9 | 6.369,5 | <0,01 |  |

| Plaquetas  (μl) | Branca | 16 | 203.222,3 | 57.144,9 | 91.218,3 | 315.226,4 | <0,01 | 19 | 251.477,7 | 65.086,9 | 123.907 | 379.048,0 | <0,01 |
| --- | --- | --- | --- | --- | --- | --- | --- | --- | --- | --- | --- | --- | --- |
|  | Parda | 47 | 194.294,0 | 32.478,0 | 130.637,1 | 257.950,9 | <0,01 | 73 | 263.923,1 | 67.775,0 | 131.084 | 396.762,1 | <0,01 |
|  | Preta | 11 | 194.750,4 | 2.4918,2 | 145.910,7 | 243.590,1 | <0,01 | 24 | 237.596,6 | 52.619,3 | 134.463 | 340.730,4 | <0,01 |

DP: desvio padrão. LI: limite inferior. LS: limite superior. *O valor total da amostra de adultos com traço falciforme é 234 participantes, porém dados faltantes não foram apresentados. **Comparação dos parâmetros de hemograma de adultos com e sem traço falciforme do estudo de Rosenfeld et al.^14^ pelo teste t de Student (diferenças estatisticamente significativas valor-p ≤ 0,05).

**Tabela S6 – Intervalos de referência de hemograma de adultos ≥ 18 anos sem traço falciforme^14^ para as séries vermelha e branca segundo raça/cor da pele e sexo, Pesquisa Nacional de Saúde, Brasil, 2014-2015**

| **Parâmetros** |  | | **Sexo Masculino** | | | | | | **Sexo Feminino** | | | | | |
| --- | --- | --- | --- | --- | --- | --- | --- | --- | --- | --- | --- | --- | --- | --- |
|  | **Raça** | | **Média** | | **LI** | | **LS** | | **Média** | | **LI** | | **LS** | |
| ***Série Vermelha*** |  | |  | |  | |  | |  | |  | |  | |
| Hemoglobina (g/dL) | Branca | |  | | -- | |  | |  | | -- | |  | |
|  | Parda | | 14,8 | | 13,0 | | 16,7 | | 13,1 | | 11,3 | | 14,8 | |
|  | Preta | |  | | -- | |  | |  | | -- | |  | |
| ***Série Branca*** | |  | |  | |  | |  | |  | |  | |  |
| Glóbulos Brancos (em mm3) | Branca | | 6.221 | | 2.960 | | 9.483 | | 6.608 | | 3.143 | | 10.074 | |
|  | Parda | | 6.093 | | 2.681 | | 9.506 | | 6.288 | | 2.772 | | 9.803 | |
|  | Preta | | 6.016 | | 3.181 | | 8.850 | | 6.165 | | 2.430 | | 9.900 | |
| Neutrófilos Absolutos (em mm3) | Branca | | 3.362 | | 651 | | 6.074 | | 3.693 | | 860 | | 6.527 | |
|  | Parda | | 3.224 | | 475 | | 5.974 | | 3.441 | | 389 | | 6.493 | |
|  | Preta | | 3.112 | | 693 | | 5.530 | | 3.221 | | 534 | | 5.908 | |

| Plaquetas (em μl) | Branca | 212.076 | 128.770 | 295.382,0 | 244.413,0 | 141.488 | 347.337,0 |
| --- | --- | --- | --- | --- | --- | --- | --- |
|  | Parda | 210.861 | 129.733 | 291.989,0 | 234.053,0 | 129.497 | 338.609,0 |
|  | Preta | 227.742 | 135.387 | 320.096,0 | 240.890,0 | 138.140 | 343.640,0 |

DP: desvio padrão. LI: limite inferior. LS: limite superior. Os valores apresentados nesta tabela foram extraídos do estudo de Rosenfeld *et al*.^14^. Foram apresentados apenas parâmetros de hemograma em que houve diferenças estatisticamente significativas (valor-p ≤ 0,05) na comparação de adultos com e sem^14^. -- Não houve diferenças estatisticamente significativas (valor-p > 0,05).
