# Supplementary material for: Differences between reference intervals of blood counts of Brazilian adults with and without sickle cell trait according to laboratory tests from the National Health Survey
Source: Rev Bras Epidemiol. 2023 Apr 21;26(Suppl 1):e230003. doi: 10.1590/1980-549720230003.supl.1 (PMC10176731; doi:10.1590/1980-549720230003.supl.1)
Supplement: Translation

# Translation: Diferenças entre intervalos de referência de hemograma de adultos brasileiros com e sem traço falciforme segundo os exames laboratoriais da Pesquisa Nacional de Saúde

**DOI:** 10.1590/1980-549720230003.supl.1.1

**Authors:** Ana Carolina Micheletti Gomide Nogueira de Sá, Alanna Gomes da Silva, Crizian Saar Gomes, Antonio Tolentino Nogueira de Sá, Deborah Carvalho Malta

**Affiliations:** I Universidade Federal de Minas Gerais, Escola de Enfermagem, Programa de Pós-Graduação em Enfermagem – Belo Horizonte (MG), Brasil.; II Universidade Federal de Minas Gerais, Escola de Enfermagem, Departamento de Enfermagem Materno Infantil e Saúde Pública, Programa de Pós-Graduação em Enfermagem – Belo Horizonte (MG), Brasil.; III Universidade Federal de Minas Gerais, Faculdade de Medicina, Programa de Pós-Graduação em Saúde Pública – Belo Horizonte (MG), Brasil.; IV Universidade Federal de Minas Gerais, Faculdade de Medicina, Programa de Pós-Graduação Ciências Aplicadas à Saúde do Adulto, Hospital das Clínicas e Departamento de Assistência à Saúde do Trabalhador – Belo Horizonte (MG), Brasil.; V Faculdade de Ciências Médicas de Minas Gerais, Departamento de Clínica Médica – Belo Horizonte (MG), Brasil.; VI Universidade Federal de Minas Gerais, Escola de Enfermagem, Departamento de Enfermagem Materno Infantil e Saúde Pública – Belo Horizonte (MG), Brasil.

**Keywords:** Inquéritos epidemiológicos, Valores de referência, Contagem de células sanguíneas, Traço falciforme, Brasil

## Abstract

**Objetivo:**

Comparar intervalos de referência (IR) de hemograma de adultos brasileiros com e sem traço falciforme (HbAS).

**Métodos:**

Estudo transversal, com a base de dados da Pesquisa Nacional de Saúde, entre 2014-2015, composta por 8.952 indivíduos; 234 adultos constituíram a amostra com HbAS. Comparou-se IR de adultos com e sem HbAS do estudo “Valores de referência para exames laboratoriais de hemograma da população adulta brasileira: Pesquisa Nacional de Saúde”, de Rosenlfed e colaboradores (2019). Utilizaram-se o método paramétrico para estabelecer os IR e o teste *t* de Student para comparação (p≤0,05).

**Resultados:**

Houve diferenças estatisticamente significativas entre IR de adultos com e sem HbAS nos homens e mulheres para hemoglobina, VCM, HCM, CHCM, glóbulos brancos, linfócitos absolutos, volume plaquetário médio e RDW; em todas as idades para glóbulos brancos e RDW nos homens e para VCM, HCM, CHCM, volume plaquetário médio e RDW nas mulheres; entre 18 a 59 anos para HCM, VCM, CHCM, neutrófilos, linfócitos e plaquetas nos homens e nas mulheres para linfócitos, glóbulos vermelhos, glóbulos brancos, neutrófilos, eosinófilos, monócitos e plaquetas; a partir de 60 anos para hemoglobina e hematócrito nos homens e nas mulheres para hematócrito, glóbulos brancos, neutrófilos e plaquetas; nas raças branca, preta e parda para glóbulos brancos, neutrófilos e plaquetas (p<0,05).

**Conclusão:**

Adultos brasileiros com HbAS tiveram menores contagens de hemoglobina, VCM, HCM, CHCM, glóbulos brancos e maiores de RDW que sem HbAS. Os resultados mostram a importância do aconselhamento genético e de pesquisas para subsidiar o manejo adequado desta condição no Brasil.

## INTRODUÇÃO

O traço falciforme (HbAS) tem sido descrito como uma condição geralmente assintomática^1,2^. A etiologia se deve à presença da hemoglobina falciforme (HbS), resultado de uma mutação em decorrência da substituição de A por T no códon 6 da cadeia beta da hemoglobina. Como consequência, tem-se a mudança do ácido glutâmico para o aminoácido valina. A hemoglobina do tipo valina leva à facilzação dos glóbulos vermelhos quando expostos a baixo limiar de oxigênio^1^. Os indivíduos afetados herdam dos pais os genes hemoglobina A (HbA) e HbS. Desta forma, são heterozigóticos possuindo um alelo anormal do gene beta da hemoglobina^1^.

Destaca-se a importância do rastreamento^3^ e aconselhamento genético nas pessoas com HbAS visando esclarecê-las quanto ao risco de ter filhos com a doença falciforme (DF), pois a herança ocorre por meio de um gene autossômico recessivo de ambos os pais^2^. Na DF, os indivíduos possuem duas cópias da variante da beta globina e a hemoglobina primária presente em seus glóbulos vermelhos (HbS), tornando-os homozigóticos (HbSS)^4^. A morbimortalidade e a gravidade da DF está associada a oclusões da microvasculatura com isquêmica tecidual, levando a crises álgicas; lesões orgânicas com asplenia funcional; vascolopatia cerebral; insuficiência renal, pulmonar e cardíaca^4,5^.

Embora o HbAS seja comumente assintomático, evidências registram casos de morte súbita^2^ nas pessoas afetadas. Isso em virtude de complicações por hipóxia, como morte súbita^6^, hematúria, hipostenúria, embolia pulmonar, infarto esplênico^2,6^, infecção urinária em mulheres, doença renal em estágio terminal, doença renal policística, infarto, acidente vascular encefálico (AVE), problemas oculares e úlceras de membros inferiores^4^.

O HbAS tem ampla distribuição continental^7^, sendo mais frequente em pessoas com ascendência africana^1,2,8^. Globalmente, estima-se que existam 300 milhões de pessoas com HbAS^1,3^, com a prevalência mais elevada na África Equatorial, Arábia, Índia, Israel, Turquia, Grécia e Itália, podendo chegar em 50% em regiões desses países^7^. No Brasil, a prevalência dessa condição varia entre 2 e 8%^7^. Estudos com dados da Pesquisa Nacional de Saúde (PNS), entre 2014 e 2015, identificou uma prevalência de 2,49% em adultos^9^.

Já a DF está entre as patologias genéticas de maior importância epidemiológica no Brasil e em outras nações^2^, considerada como um problema de saúde pública pela Organização Mundial de Saúde (OMS)^3^. Mundialmente, em 2010, a DF afetou 305.800 recém-nascidos, e espera-se um aumento para 404.200 em 2050^10^. No Brasil, a prevalência varia de 1,1 a 9,8% e de 0,8 a 60 por 100.000 nascidos vivos em diferentes regiões do país^2^.

Alterações nos parâmetros hematológicos foram identificadas em pessoas com DF^8,11^. Nas pessoas com HbAS, apesar de ser descrito que geralmente os parâmetros hematológicos se apresentam normais ou sem alterações laboratoriais importantes^4^, nos processos patológicos que causam hipóxia, acidose, desidratação, hiperosmolalidade ou hipotermia, essa condição pode levar à vaso-oclusão devido a eritrócitos rígidos^11^.

Diferenças nos parâmetros hematológicos de pessoas com HbAS foram encontradas em populações de outros países, o que denota que os intervalos de referência (IR) de hemograma podem sofrer influências geográficas e étnico-racionais^3,8^. Entretanto, estudos sobre o perfil hematológico do HbAS no Brasil são escassos. Esse estudo avança ao fornecer de forma inédita, pelos exames da PNS, os parâmetros hematológicos de adultos com HbAS e as diferenças encontradas em relação aos parâmetros de adultos sem HbAS no país. Os achados podem contribuir para subsidiar o manejo do HbAS no Brasil.

Desta forma, o presente estudo teve como objetivo comparar os IR de hemograma de adultos brasileiros com e sem HbAS.

## MÉTODOS

### Desenho do estudo

Estudo transversal com dados dos exames laboratoriais da PNS, entre 2014 e 2015.

### Contexto e fonte dos dados

A PNS é uma pesquisa nacional e de base domiciliar executada pelo Instituto Brasileiro de Geografia e Estatística (IBGE), em parceria com Ministério da Saúde (MS)^12^.

A PNS edição de 2013 utilizou amostra probabilística por conglomerados em três estágios, com estratificação das unidades primárias de amostragem (UPA), sendo selecionados no primeiro, segundo e terceiro estágios, respectivamente: setores censitários; 10 a 14 domicílios em cada UPA; e um morador com idade a partir de 18 anos. Em todos os estágios, os processos de seleção foram feitos por amostragem aleatória simples (AAS). Foram obtidos registros de 64.348 domicílios, sendo 60.202 entrevistas em adultos. Ademais, a coleta de exames foi planejada em subamostra de 25% dos setores censitários e foram coletados, entre 2014 e 2015, em 8.952 adultos^12^.

Devido ao desenho complexo de amostragem da PNS e as probabilidades desiguais de seleção, foram utilizados pesos amostrais^12^. Para o uso da base de dados do laboratório da PNS, as ponderações foram calculadas por procedimentos de pós-estratificação por sexo, faixa de idade, raça/cor e grau de escolaridade segundo grande região, a partir da amostra total da pesquisa^12^.

As coletas de exames laboratoriais foram realizadas a qualquer hora do dia e sem jejum. Para coleta de hemograma e eletroforese de hemoglobina foram utilizados tubos com ácido etilenodiamino tetra-acético (EDTA). As amostras foram analisadas por meio do analisador automático de células^12^. A pesquisa de hemoglobinopatias foi feita pelo método HPLC, e os resultados dos exames individuais foram interpretados fornecendo os parâmetros normais, homozigotos ou heterozigotos para Hemoglobina S (HbS), Hemoglobina C (HbC), Hemoglobina D (HbD) e outras eventuais hemoglobinopatias^9^. Maiores detalhes metodológicos estão disponíveis em outras publicações^12,13^.

A base de dados e questionários da PNS utilizados neste estudo encontram-se disponíveis em: www.pns.fiocruz.br.

### Participantes

Os participantes foram adultos a partir de 18 anos. Utilizou-se a base de dados da PNS composta por 8.952 indivíduos. Foram excluídos 330 adultos em razão da falta de resultados de exames, material insuficiente, perda da amostra e hemólise^9^. Neste estudo, foram analisados dados de 234 adultos com HbAS. Os dados analisados de adultos sem HbAS foram extraídos do estudo de Rosenfeld et al.^14^.

### Intervalos de referência

#### Intervalos de adultos com traço falciforme:

Os IR dos parâmetros de hemograma (descritos a seguir) de adultos com HbAS foram estimados considerando-se o limite inferior (LI) como a média −1,96 DP (desvio padrão) e o limite superior (LS), a média +1,96 DP (desvio padrão).

#### Intervalos de adultos sem HbAS

Para adultos sem HbAS, foram utilizados os valores das médias e dos IR (LI e LS) dos parâmetros de hemograma do estudo de Rosenfeld et al.^14^.

### Variáveis

As variáveis foram: sociodemográficas: sexo (masculino; feminino); idade (faixa etária em anos: 18 a 59; 60 ou mais); raça/cor (branca; parda; preta). Presença de HbAS: (sim; não). Parâmetros de hemograma: série vermelha: glóbulos vermelhos (milhões/mm^3^); hemoglobina (g/dL); hematócrito (%); volume corpuscular médio (VCM) (fL); hemoglobina corpuscular média (HCM) (pg); concentração de hemoglobina corpuscular média (CHCM) (g/dL); amplitude de distribuição dos eritrócitos (RDW) (%). Série branca: glóbulos brancos (mm^3^); neutrófilos absolutos (mm^3^); eosinófilos absolutos (mm^3^); basófilos absolutos (mm^3^); linfócitos absolutos (mm^3^); monócitos absolutos (mm^3^); plaquetas (μl); volume plaquetário médio (fL).

### Análises estatísticas

Os IR foram estimados pelo método paramétrico. A amostra foi particionada segundo sexo, idade e raça/cor da pele. Para cada partição foram calculadas as médias, DP e os IR ligados aos LI e LS (média ± 1,96 DP), segundo sexo, faixa etária e raça/cor. Em virtude da amostra da PNS ser suficiente grande e se aproximar da distribuição normal, utilizou-se o teste t de Student para comparar IR de adultos com e sem HbAS do estudo de Rosenfeld et al.^14^. O nível de significância adotado foi de 5%.

As análises dos dados foram realizadas no software *Data Analysis and Statistical Software (Stata)*, versão 14, utilizando-se o conjunto de comandos para análise do módulo survey, que considera os pesos de pós-estratificação.

### Aspectos éticos

A PNS foi aprovada pela Comissão Nacional de Ética em Pesquisa do Conselho Nacional de Saúde (parecer n° 328.159). A participação do adulto foi voluntária, e a confidencialidade das informações garantida^13^.

## RESULTADOS

Para a série vermelha, houve diferenças estatisticamente significativas para alguns parâmetros do hemograma ao se comparar IR de adultos com HbAS e sem HbAS^14^ (p≤0,05) (Tabela 1). Homens e mulheres com HbAS apresentaram valores médios mais baixos de hemoglobina, VCM, HCM, CHCM (Tabela 1) quando comparados aos sem HbAS^14^, conforme nota-se pelos valores das médias e dos IR do estudo de Roselfeld et al.^14^ dispostos na Tabela S1. Para RDW, as médias de homens e mulheres com HbAS foram mais elevadas que nos sem HbA^14^. Homens com HbAS ainda apresentaram médias menores de hematócrito que os sem HbAS^14^ (Tabelas 1 e S1).

**Tabela 1:** Intervalos de referência de hemograma de adultos ≥18 anos com traço falciforme para série vermelha segundo sexo, Pesquisa Nacional de Saúde, Brasil, 2014–2015.

Parâmetros	Sexo	n*	Média	DP	LI	LS	p†
Glóbulos vermelhos (milhões/mm^3^)	Masculino	84	5,1	0,4	4,3	5,9	0,6441
	Feminino	128	4,6	0,4	3,8	5,4	0,05
Hemoglobina (g/dL)	Masculino	84	14,5	1,3	12,0	17,0	0,0013
	Feminino	128	13,0	1,5	10,1	15,9	0,0004
Hematócrito (%)	Masculino	84	45,5	4,1	37,5	53,5	0,0254
	Feminino	128	40,5	3,6	33,4	47,6	0,0652
Volume corpuscular médio (fL)	Masculino	84	89,6	4,9	80	99,2	0,0027
	Feminino	128	88,0	5,1	78	98	<0,01
Hemoglobina corpuscular média (pg)	Masculino	84	28,9	1,7	25,6	32,2	<0,01
	Feminino	128	28,3	1,7	25,0	31,5	<0,01
Concentração de hemoglobina corpuscular média (g/dL)	Masculino	84	32,2	1,1	30,0	34,4	0,0029
	Feminino	128	32,1	1,0	30,1	34,0	0,0007
Amplitude de distribuição dos eritrócitos (RDW) (%)	Masculino	84	13,9	1,0	11,9	15,8	0,0001
	Feminino	128	14,3	1,4	11,7	17,0	<0,01

DP: desvio padrão; LI: limite inferior; LS: limite superior.

* O valor total da amostra de adultos com traço falciforme é 234 participantes, porém dados faltantes não foram apresentados;

† Comparação dos parâmetros de hemograma de adultos com e sem traço falciforme do estudo de Rosenfeld et al.^14^ pelo teste *t* de Student (diferenças estatisticamente significativas valor-p≤0,05).

Com relação à série branca, para ambos os sexos observam-se diferenças estatisticamente significativas nos IR de alguns parâmetros do hemograma de homens e mulheres com HbAS comparados aos sem HbAS^14^ (p≤0,05) (Tabela 2). Foram mais baixas as médias de glóbulos brancos, neutrófilos, linfócitos e volume plaquetário médio em homens e mulheres com HbAS que nos sem HbAS^14^. Mulheres com HbAS também tiveram médias mais elevadas de eosinófilos e plaquetas que as sem HbAS^14^ e homens com HbAS apresentaram médias mais baixas de plaquetas que os sem HbAS^14^ (Tabelas 2 e S1).

**Tabela 2:** Intervalos de referência de hemograma de adultos ≥ 18 anos com traço falciforme para a série branca segundo sexo, Pesquisa Nacional de Saúde, Brasil, 2014-2015.

Parâmetros	Sexo	n*	Média	DP	LI	LS	p†
Glóbulos brancos (mm^3^)	Masculino	75	5.374,8	1.682,2	2.077,7	8.671,9	<0,01
	Feminino	117	6.390,2	2.056,7	2.359,1	10.421,3	<0,01
Neutrófilos absolutos (mm^3^)	Masculino	74	2.940,4	1.373,1	249,1	5.631,7	<0,01
	Feminino	117	3.674,9	1.723,7	296,4	7.053,4	<0,01
Eosinófilos absolutos (mm^3^)	Masculino	74	304,4	274,4	233,4	842,2	0,3509
	Feminino	117	225,0	214,6	195,6	645,6	0,0189
Basófilos absolutos (mm^3^)	Masculino	74	25,5	23,4	20,3	71,4	0,1260
	Feminino	117	33,5	31,4	28,0	95,0	0,9750
Linfócitos absolutos (mm^3^)	Masculino	74	1.702,3	669,3	309,5	3.014,1	<0,01
	Feminino	117	2.074,4	756,9	590,9	3.557,9	<0,01
Monócitos absolutos (mm^3^)	Masculino	74	428,0	232,6	55,8	800,1	0,8392
	Feminino	117	382,5	191,6	7,0	758,0	0,0518
Plaquetas (μl)	Masculino	64	197.645,2	42.288,1	114.760,5	280.529,9	<0,01
	Feminino	119	255.814,0	64.357,9	129.672,5	381.955,5	<0,01
Volume plaquetário médio (fL)	Masculino	64	8,8	4,1	0,8	16,8	0,042
	Feminino	119	8,6	3,8	1,2	16,0	<0,01

DP: desvio padrão; LI: limite inferior; LS: limite superior.

* O valor total da amostra de adultos com traço falciforme é 234 participantes, porém dados faltantes não foram apresentados;

† Comparação dos parâmetros de hemograma de adultos com e sem traço falciforme do estudo de Rosenfeld et al.^14^ pelo teste *t* de Student (diferenças estatisticamente significativas valor-p≤0,05).

Com relação à idade, para a série vermelha, nota-se diferenças estatisticamente significativas nos IR de homens com HbAS comparados aos sem HbAS^14^ (p≤0,05) (Tabela S2). As médias foram menores entre 18 e 59 anos para HCM, VCM e CHCM, e a partir de 60 anos para hemoglobina, hematócrito e HCM nos homens com HbAS do que nos sem HbAS^14^. Houve maiores médias de RDW nos homens entre 18 e 59 anos com HbAS que nos sem HbAS^14^. As médias de hemoglobina, VCM, HCM e CHCM em mulheres entre 18 e 59 anos e 60 anos ou mais foram menores nas com HbAS que nas sem HbAS^14^. Mulheres com HbAS a partir de 60 anos ou mais tiveram menores médias de hematócrito do que as sem HbAS^14^. Maiores médias de glóbulos vermelhos e RDW foram encontrados em mulheres entre 18 e 59 anos com HbAS em comparação com as sem HbAS^14^ (Tabelas S2 e S3).

Para a série branca, houve diferenças estatisticamente significativas nos IR de adultos com e sem HbAS^14^ ao se estratificar por idade (p≤0,05) (Tabela S4). Os valores médios de glóbulos brancos foram menores nos homens com HbAS que nos sem HbAS^14^ entre 18 e 59 e aos 60 anos ou mais. Foram identificadas menores médias de neutrófilos, linfócitos, plaquetas e volume plaquetário médio nos homens com HbAS quando comparados aos sem HbAS^14^ nas duas faixas etárias estudadas. Para mulheres com HbAS, foram menores as médias de volume plaquetário médio nas duas faixas etárias do que nas sem HbAS^14^, e a partir dos 60 anos, mulheres com HbAS tiveram menores médias de glóbulos brancos, neutrófilos e plaquetas do que as sem HbAS^14^. Também nas mulheres com HbAS em relação às sem HbAS^14^, foram encontradas maiores médias de glóbulos brancos, neutrófilos, eosinófilos, monócitos e plaquetas entre 18 e 59 anos (p≤0,05) (Tabela S3 e S4).

Houve diferenças ao se comparar os IR de alguns parâmetros de hemograma entre homens e mulheres com e sem HbAS^14^ da raça/cor branca, parda e preta para hemoglobina, glóbulos brancos, neutrófilos e plaquetas (p≤0,05) (Tabela S5). As médias foram levemente menores em ambos os sexos para hemoglobina na raça parda nos adultos com HbAS que nos sem HbAS^14^. Nos homens das raças branca, preta e parda com HbAS, foram menores as médias de glóbulos brancos, neutrófilos e plaquetas do que nos sem HbAS. Mulheres com HbAS da raça branca tiveram menores médias de glóbulos brancos e neutrófilos que as sem HbAS^14^, assim como as da raça preta para plaquetas. Nas mulheres com HbAS das raças preta e parda, foram encontrados maiores valores médios de glóbulos brancos e neutrófilos, e nas das raças branca e parda maiores valores médios de plaquetas que nas sem HbAS^14^ (Tabelas S5 e S6).

## DISCUSSÃO

Este estudo analisou pela primeira vez, por meio dos dados da PNS laboratorial, as diferenças de IR hematológicos de adultos brasileiros com e sem HbAS^14^. Os achados mostram menores contagens para hemoglobina, VCM, HCM, CHCM, glóbulos brancos, linfócitos e volume plaquetário médio e maiores contagens para RDW nos adultos com HbAS comparados aos sem HbAS^14^, para ambos os sexos. Ao se estratificar segundo sexo e idade, foram identificadas diferenças em alguns valores parâmetros de séries branca e vermelha quando se comparou adultos com HbAS e sem HbAS^14^. Ademais, foram encontradas diferenças nos IR de adultos com HbAS das raças/cor de pele branca, preta e parda para glóbulos brancos, neutrófilos e plaquetas, e ainda na raça parda para hemoglobina, quando comparados aos sem HbAS^14^. Tais achados refletem a relevância de se conhecer o perfil hematológico de pessoas com HbAS para o alcance do manejo adequado dessa condição no país.

Tendo em vista que tem sido descrito que parâmetros hematológicos de pessoas com HbAS geralmente não apresentam alterações laboratoriais importantes^4^, as diferenças encontradas no presente estudo, em IR de alguns parâmetros do hemograma de adultos brasileiros com e sem HbAS^14^, evidenciam a necessidade de mais estudos para melhor compreensão das implicações clínicas nas pessoas com essa condição.

Neste estudo, os resultados encontrados de IR de hemograma em adultos com e sem HbAS^14^, segundo sexo, foram consonantes com estudo em Gana, porém em que foram comparados indivíduos saudáveis e com DF^8^. Com relação à série vermelha do hemograma, nos adultos brasileiros de ambos os sexos com HbAS, os valores alterados de hemoglobina, CHCM e VCM indicam a presença de anemia^15–18^, microcitose^17–19^ e hipocromia^16,17^, diferentemente dos adultos sem HbAS, cujos valores de referência encontraram-se sem alterações^14^. Além disso, os baixos valores de CHCM nos adultos com HbAS, possivelmente, podem estar associados a anemia ferropriva^20^ ou a talassemias^21^. Outros achados nessas pessoas com HbAS se referem à anemia microcítica hipocrômica com RDW elevado, que também é característica da falta de ferro, por déficit nutricional, deficiência na absorção por alterações gastrointestinais e perda crônica desse micronutriente^22^, evidenciado pelos LS de RDW.

Tal achado é alarmante pela possibilidade de má nutrição em pessoas com HbAS no Brasil, e ainda mais levando-se em consideração que a hemólise crônica resulta em uma maior disponibilidade de ferro, sendo a sua deficiência pouco provável na DF^23,24^. Isso evidencia a necessidade de intensificação das ações no campo da segurança alimentar no Brasil, com atenção aos grupos mais vulneráveis^16^. Situação agravada pela pandemia de COVID-19 que recolocou o Brasil no mapa da fome^25^, podendo ter contribuído para piora deste cenário, tanto nas pessoas com HbAS como com DF. Também em cumprimento às pactuações feitas pelo Brasil nas esferas nacionais e internacionais, de redução da anemia por carência de ferro até 2030, como uma de suas prioridades em saúde públicas^16^.

As análises do hemograma por idade seguiram padrões semelhantes às análises por sexo, ocorrendo em homens e mulheres com HbAS de todas idades, quando comparados aos sem HbAS^14^, alterações nos IR de VCM, HCM e CHCM. Possíveis explicações se devem às condições de acesso e assistência à saúde e alimentação, pois a variabilidade clínica da DF é influenciada por fatores sociais e econômicos^26^, o que também pode estar acontecendo no HbAS. Outras possíveis explicações para os valores mais baixos de VCM, HCM e CHCM, observados nos adultos com HbAS em relação aos sem HbAS, são decorrentes dos efeitos de anemia de doença crônica, maior risco de infecções e do grau hemólise em que tais parâmetros encontram-se reduzidos^24^.

Ao se analisar a série branca do hemograma segundo sexo, com relação aos glóbulos brancos e neutrófilos, o que chama a atenção são os valores médios ainda mais baixos encontrados nos adultos brasileiros com HbAS comparados aos sem HbAS. Nas análises por idade, nota-se a presença de leucopenia^27^ pelos LI em todas as faixas etárias e em ambos os sexos, e a presença de leucocitose^27^ em mulheres entre 18 e 59 anos identificada pelos LS. A literatura descreve que o declínio de leucócitos está relacionado ao aumento da idade em decorrência do desenvolvimento da resposta imune na medida em que ocorre a exposição a patógenos^28,29^. A leucopenia também pode acontecer em virtude de infecções virais e bacterianas e uso de medicamentos, e é intercambiável com neutropenia^30^, como pode-se observar pelos LI de neutrófilos encontrados em homens e mesmo nas mulheres brasileiras com HbAS, cujos valores médios foram pouco mais elevados que os sem HbAS^14^. O manejo dessas situações requer identificação da causa e terapia eficaz^30^. Tal achado possivelmente denota maior fragilidade clínica dos adultos brasileiros com HbAS expostos à desnutrição e a infecções.

Quanto a maior contagem de glóbulos brancos em mulheres brasileiras entre 18 e 59 anos com HbAS, quando comparado nas sem HbAS^14^ identificado pelos LS, resultados semelhantes foram encontrados na Nigéria, mas na DF^24^. Também cabe mencionar que, nas pessoas com DF, a leucocitose pode acontecer por conta da autoesplenectomia resultante da oclusão dos vasos esplênicos, aumentando a vulnerabilidade a infecções^24^. A leucocitose na DF denota mau prognóstico, sendo fator de risco de morte precoce, AVE e síndrome torácica aguda, enquanto que a redução da contagem de neutrófilos está associada a bom prognóstico^24^. Há evidências que sugerem que a função fagocitária dos neutrófilos pode ser reduzida em indivíduos com DF, implicando que sua competência fagocítica afeta a gravidade clínica da doença^24,31^. Todas as explicações carecem de evidências empíricas e teóricas, portanto necessitam ser melhor investigadas em adultos com HbAS.

Quanto às diferenças significativas encontradas nas plaquetas, ao se estratificar por sexo e idade, os maiores valores médios nas mulheres entre 18 e 59 anos com HbAS em relação aos sem HbAS^14^ podem aparecer devido a trombocitose, atribuído à anemia hemolítica e à autoesplenectomia associada à DF^24,32^. Na literatura está bem estabelecido que a trombocitose está associada à DF e a vários tipos de anemia^24,32^. Já os menores níveis médios de plaquetas em homens com HbAS comparados aos sem HbAS^14^, entre 18 e 59 anos, supostamente se deve às alterações em componentes da hemostasia, incluindo função plaquetária, mecanismos pro-coagulantes, anticoagulantes e sistema fibrinolítico na DF^33^. O estado hipercoagulável presente em pessoas com HbS se relaciona com alterações qualitativas de plaquetas^33^, o que possivelmente explicaria esses achados.

As demais alterações hematológicas encontradas em adultos com HbAS quando comparados aos sem HbAS^14^, nas mulheres entre 18 e 59 anos para eosinófilos e monócitos, e de hematócrito a partir dos 60 anos, bem como as encontradas em homens de 18 a 59 anos para linfócitos e hemoglobina, e hematócrito aos 60 anos ou mais, podem ser atribuídas a fatores comportamentais, genéticos, nutricionais, socioeconômicos, exposição a alérgenos, infecções e cargas parasitárias^34^.

No presente estudo, houve diferenças substanciais nos IR hematológicos de adultos brasileiros com e sem HbAS^14^ segundo raça. A anemia identificada por menores níveis de hemoglobina na raça parda em adultos com HbAS em relação aos sem HbAS^14^ é algo que merece ser investigado, haja vista que condições associadas à anemia diferem-se por raça^35^. Embora diferenças raciais em parâmetros hematológicos possam ser decorrentes de hereditariedade e doenças crônicas, fatores socioeconômicos diferem de acordo com a raça^35^. A literatura registra a escassez de estudos que exploram as diferenças raciais de parâmetros hematológicos^35^. As populações negras são sub-representadas em estudos epidemiológicos sobre a temática^35^, e também identificamos a carência de investigações na população parda. Esse é um ponto importante de discussão, haja vista que a população brasileira se caracteriza pela miscigenação de raças, etnias e segmentos sociais e econômicos^14^, o que possui implicações na epidemiologia do HbAS e da DF no país^36^.

Este estudo teve limitações inerentes aos estudos transversais, como a impossibilidade de estabelecer relação causal; alguns achados nos parâmetros de hemograma podem ser reflexos dos estilos de vida e tratamento. Contudo, o perfil hematológico de pacientes com HbAS em brasileiros não foi estudado extensivamente nos últimos anos, sendo este o primeiro estudo com esse propósito. Dado a amostra representativa da população brasileira, a generalização dos resultados é relativamente segura para estimativas nacionais. Não foi possível identificar pessoas com DF por indisponibilidade na base de dados da PNS; as alterações laboratoriais podem remeter ao subdiagnóstico da DF no país. Assim, os resultados devem ser interpretados com prudência. Ademais, há controvérsias na literatura se o HbAS é um estado benigno ou um fenótipo intermediário da doença, e paralelamente tem-se a carência acentuada de estudos sobre essa condição e suas complicações, as quais podem estar sendo subestimadas^37^. Evidências sugerem que o HbAS pode não ser um estado completamente benigno e nem uma doença, mas um fator de risco para eventos adversos que resultam da interação entre influências genéticas e ambientais^38^.

Este estudo evidenciou a existência de diferenças entre os IR de adultos brasileiros com e sem HbAS^14^. Esses resultados mostram a importância da triagem neonatal para identificação de pessoas HbAS, aconselhamento genético e a necessidade de pesquisas que caracterizem as consequências e riscos à saúde, para subsidiar o controle e a prevenção de agravos. A detecção de indivíduos com hemoglobinopatias heterozigóticas como a HbAS é extremamente relevante em termos de saúde pública^39^ para o manejo adequado no Brasil.
